# Role of Sarcoplasmic Reticulum Calcium in Development of Secondary Calcium Rise and Early Afterdepolarizations in Long QT Syndrome Rabbit Model

**DOI:** 10.1371/journal.pone.0123868

**Published:** 2015-04-13

**Authors:** Po-Cheng Chang, Hung-Ta Wo, Hui-Ling Lee, Shien-Fong Lin, Ming-Shien Wen, Yen Chu, San-Jou Yeh, Chung-Chuan Chou

**Affiliations:** 1 Division of Cardiology, Department of Internal Medicine, Chang Gung Memorial Hospital, Linko, Taoyuan, Taiwan; 2 Chang Gung University College of Medicine, Taoyuan, Taiwan; 3 Department of Anesthesia, Chang Gung Memorial Hospital, Taipei, Taiwan; 4 Institute of Biomedical Engineering, National Chiao Tung University, Hsin Chu, Taiwan; 5 Division of Thoracic Surgery, Chang Gung Memorial Hospital, Linko, Taoyuan, Taiwan; 6 Institute of Biomedical Engineering, National Chiao Tung University, Hsin Chu, Taiwan; Cinvestav-IPN, MEXICO

## Abstract

**Background:**

L-type calcium current reactivation plays an important role in development of early afterdepolarizations (EADs) and torsades de pointes (TdP). Secondary intracellular calcium (Ca_i_) rise is associated with initiation of EADs.

**Objective:**

To test whether inhibition of sarcoplasmic reticulum (SR) Ca^2+^ cycling suppresses secondary Ca_i_ rise and genesis of EADs.

**Methods:**

Langendorff perfusion and dual voltage and Ca_i_ optical mapping were conducted in 10 rabbit hearts. Atrioventricular block (AVB) was created by radiofrequency ablation. After baseline studies, E4031, SR Ca^2+^ cycling inhibitors (ryanodine plus thapsigargin) and nifedipine were then administrated subsequently, and the protocols were repeated.

**Results:**

At baseline, there was no spontaneous or pacing-induced TdP. After E4031 administration, action potential duration (APD) was significantly prolonged and the amplitude of secondary Ca_i_ rise was enhanced, and 7 (70%) rabbits developed spontaneous or pacing-induced TdP. In the presence of ryanodine plus thapsigargin, TdP inducibility was significantly reduced (2 hearts, 20%, p = 0.03). Although APD was significantly prolonged (from 298 ± 30 ms to 457 ± 75 ms at pacing cycle length of 1000 m, p = 0.007) by ryanodine plus thapsigargin, the secondary Ca_i_ rise was suppressed (from 8.8 ± 2.6% to 1.2 ± 0.9%, p = 0.02). Nifedipine inhibited TdP inducibility in all rabbit hearts.

**Conclusion:**

In this AVB and long QT rabbit model, inhibition of SR Ca^2+^ cycyling reduces the inducibility of TdP. The mechanism might be suppression of secondary Ca_i_ rise and genesis of EADs.

## Introduction

Drug-induced acquired long QT syndrome and torsades de pointes (TdP) is one of the most serious adverse effects of medications. There is close relationship between risk of prolonged QT interval/TdP and inhibition of rapidly activating delayed rectifier potassium current (I_Kr_) [1,2]. I_Kr_ is encoded by the hERG gene, which is the gene encoding mutation in long QT syndrome type 2 [3]. However, the risk of TdP development is not always linked to prolonged QT interval. Amiodarone has been wildly used for ventricular and supraventricular tachyarrhythmias, especially in patients with heart failure. Although it causes significant QT prolongation, the risk of provoking TdP is relatively minor. The relationship between the risk of TdP and the severity of QT prolongation remains mysterious.

Early afterdepolarizations (EADs) are secondary depolarization during repolarization of action potential and usually developed in the presence of prolonged action potential duration (APD). L-type calcium current (I_Ca,L_) reactivation is required in the initiation of EADs [4]. Previously we observed secondary intracellular calcium (Ca_i_) rise in a rabbit heart failure model [5]. It has also been reported that I_Ca,L_ blockade abolished EAD development in heart failure animal models [6]. Whether or not sarcoplasmic reticulum (SR) Ca^2+^cycling also plays a role in genesis of EADs and secondary Ca^2+^ rise is still unclear. In this study, we hypothesized that SR Ca^2+^ cycling inhibition suppressed secondary Ca_i_ rise and development of EADs. We used a long QT syndrome rabbit model with atrioventricular block (AVB) creation and E4031 containing low-K^+^-low-Mg^2+^ Tyrode’s solution perfused to test the hypothesis. This study followed the previous studies of long QT rabbit models [7,8]. E4031 is a specific I_Kr_ inhibitor and has been used for creation of long QT animal model.

## Materials and Methods

The research protocol was approved by the Institutional Animal Care and Use Committee (IACUC) of Chang Gung Memorial Hospital (Permit Number: 2012121704) and conformed to the Guide for Use of Laboratory Animals. All surgery was performed under general anesthesia with ketamine, rompun and isoflurane, and all efforts were made to minimize suffering. Ten adult New Zealand white rabbits (2.5–3.5 kg) were used in this study.

### Optical mapping and AVB model

We created AVB after harvesting rabbit hearts in this study using a modified atrioventricular node ablation method [5] and the same optical mapping method described previously [9,10]. In brief, the rabbits were generally anesthetized with intravenous injection of ketamine (8 mg/kg) and xylazine (8mg/kg).When the rabbits were fully anesthetized and unresponsive to physical stimuli, the hearts were rapidly harvested and Langendorff-perfused with 37°C standard Tyrode’s solution of the following composition: 125 mM NaCl, 4.5 mM KCl, 0.5 mM MgCl_2_, 24 mM NaHCO_3_, 1.8 mM NaH_2_PO_4_, 1.8 mM CaCl_2_, 5.5 mM glucose and 100 mg/L albumin, equilibrated with 95% O_2_ and 5% CO_2_ in de-ionized water and with a pH of 7.40. We then performed radiofrequency ablation to create AVB using a 7Fr. quadripolar large-tip ablation catheter (Biosense Webster, Diamond Bar, CA, USA) at a output energy of 10~20W generated by a radiofrequency generator (Radionics RFG-3C, Radionics Inc., MA, USA). The target ventricular escape rate after AV node ablation was less than 60 beats per minute.

The hearts were stained with Rhod-2-AM (Molecular Probes, Carlsbad, CA, USA) for Ca_i_ and RH-237 (Molecular Probes) for membrane potential (V_m_). We used a laser light at a wavelength of 532 nm (Millennia, Spectra-Physics Inc., Santa Clara, CA, USA) to excite the fluorescence dyes. The emitted fluorescence was acquired and filtered (715 mm for V_m_ and 580 nm for Ca_i_) with twocharge-coupled device cameras (CA-D1-0128T, Dalsa Inc., Waterloo, Ontario, Canada) at 4 ms/frame temporal resolution and 128 x 128 pixels with spatial resolution of 0.35 x 0.35 mm^2^ per pixel. To provoke development of TdP, the perfused solution was changed to a modified low-K^+^ (2.25 mM)/low Mg^2+^ (0.25 mM) Tyrode’s solution after fluorescence dye staining. Motion artifacts were suppressed by blebbistatin (15 μmol/L, Tocris Bioscience, Minneapolis, MN, USA).

### Experimental protocol

Pseudo-electrocardiography (pECG) was recorded using three electrodes placed at the left atrium, the posterior wall of the left ventricle and the right ventricle. A bipolar catheter was inserted into the right ventricular apex for pacing at twice threshold. Because of significantly prolonged APD in this long-QT syndrome model, the optical mapping signals were acquired at cycle lengths of 1000, 800, 600, 500, 400, 350, 300 ms and then down to the shortest 1:1 captured cycle length with a 20 ms step. A S1/S2/S3 short-long-short pacing protocol (S1 30 beats with S1-S1 500 ms, a long S1-S2 of 1000 or 2000 ms and a S2-S3 starting from 500 ms and gradually shortened to the ventricular effective refractory period) was used to induce TdP. Some TdP episodes were also induced by a short burst pacing following a long pause in this AVB model. After the baseline studies, E4031 (0.5 μM), ryanodine (1.0 μM) plus thapsigargin (1.0 μM), and nifedipine (2.0 μM) were administered subsequently, and the experimental protocols were repeated after each set of medications.

### Data analysis

APD_80_ was measured at the level of 80% repolarization of action potential. Secondary rise of Ca_i_ is defined as the spontaneous increase of the Ca_i_ at the downslope of the primary Ca_i_ released. The amplitude of secondary Ca_i_ rises is defined as the largest deviation from a line drawn between the onset and offset of the secondary Ca_i_ rise as previously described [5]. TdP was defined as polymorphic ventricular tachycardia more than 3 beats. Continuous variables with normal distribution were expressed as the mean ± SEM, and categorical variables were expressed as number (percentage). Differences in continuous variables between different zones of the same heart with normal distribution were analyzed by paired t-test. One-way repeated measures ANOVA with post-hoc LSD analysis was used to compare continuous variables in the presence of different sets of medications with baseline values. The differences in categorical variables at different concentrations were tested using Cochran’s Q test. Differences were considered significant when the probability value was < 0.05.

## Results

### Inducibility of TdP in AVB model

**Fig 1:**
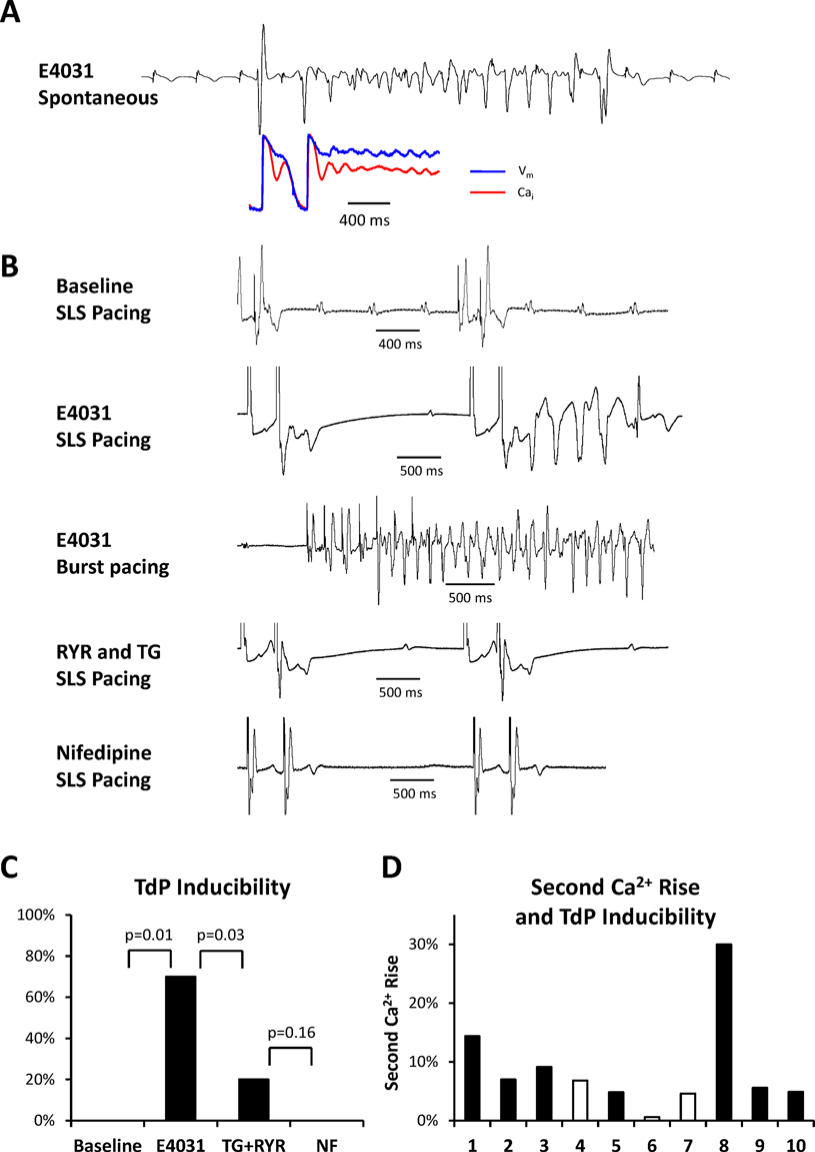
TdP and EAD inducibility. **A.** A representative example of spontaneous TdP ventricular tachycardia and the V_m_-Ca_i_ traces. **B.** Short-long-short (SLS) pacing did not induce TdP ventricular tachycardia at baseline (the top subpanel). In the presence of E4031, rabbit hearts developed pacing-induced TdP (the second and third subpanels). After ryanodine plus thapsigargin administration, only 2 (20%) hearts developed TdP (the forth subpanel). Nifedipine abolished the inducibility of TdP (the bottom subpanel). **C.** Inducibility of TdP inducibility at baseline, after administration of E4031, ryanodine plus thapsigargin and nifedipine. **D.** The relationship between amplitude of secondary Ca_i_ rise and TdP inducibility of each heart (numbers indicate the serial number of rabbit hearts). Filled bars indicate hearts with inducible TdP and unfilled bars are hearts without inducible TdP. Note the 3 hearts without TdP inducibility had relatively lower secondary Ca_i_ rise.

We successfully obtained optical mapping recording in 10 rabbit hearts. There was no inducible TdP or EADs at baseline. We recorded spontaneous or pacing-induced TdP in 7 hearts (70%) in the presence of E4031. Fig 1 shows representative examples of spontaneous (panel A) and pacing-induced TdP (panel B, the second and the third subpanels). Panel A shows secondary Ca_i_ rise and Ca_i_ oscillation (red trace) during the spontaneous TdP. After ryanodine plus thapsigargin administration, the inducibility of TdP was significantly suppressed (Fig 1B, the forth subpanel) and only 2 hearts had inducible TdP in the presence of ryanodine plus thapsigargin (Fig 1C, 20%, p = 0.03). We further applied nifedipine and the inducibility of TdP was completely suppressed (0%). The bottom subpanel of Fig 1B shows a representative example in the presence of nifedipine. The results were compatible with previous findings that SR Ca^2+^ cycling also played an important role in the genesis of EADs.

### Action potential duration

**Fig 2:**
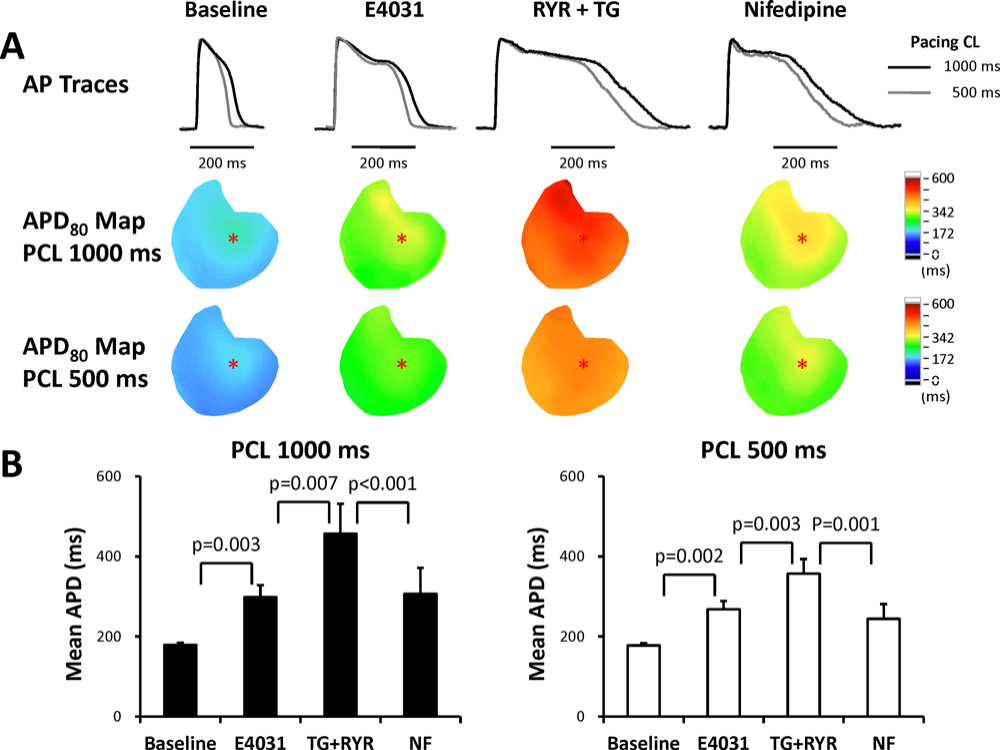
APD. **A.** Representative action potential traces and APD maps at pacing cycle lengths of 1000 ms and 500 ms. **B.** Mean APD_80_ at pacing cycle lengths of 1000 ms and 500 ms.

Fig 2A shows representative action potential traces and APD maps at baseline, in the presence of E4031, further administration of ryanodine plus thapsigargin, and then nifedipine, respectively. APD_80_ was longer in the presence of E4031 and was further prolonged after administration of ryanodine plus thapsigargin at both 1000 ms and 500 ms pacing cycle lengths (PCLs). Nifedipine shortened APD_80_. Because of significantly prolonged APD_80_, we were not able to pace the hearts at pacing cycles shorter than 400 ms after applying E4031 in most of the hearts. Statistical tests showed that E4031 significantly prolonged APD_80_ (from 179 ± 5 ms to 298 ± 30 ms, p = 0.003, and from 178 ± 6 ms to 267 ± 21 ms, p = 0.002, at PCL 1000 ms and 500 ms, respectively, Fig 2B), and ryanodine plus thapsigargin further prolonged APD_80_ (457 ± 75 ms and 357 ± 637 ms, at PCLs of 1000 ms and 500 ms, p = 0.007 and p = 0.003, respectively). Nifedipine significantly shortened the extremely prolonged APD_80_ (307 ± 65 ms and 244 ± 37 ms, at PCLs of 1000 ms and 500 ms, p < 0.001 and p = 0.001, respectively). Although inhibition of SR Ca^2+^ cycling further prolonged APD_80_, the inducibility of TdP was suppressed as shown in Fig 1C.

### Secondary Ca_i_ rise

**Fig 3:**
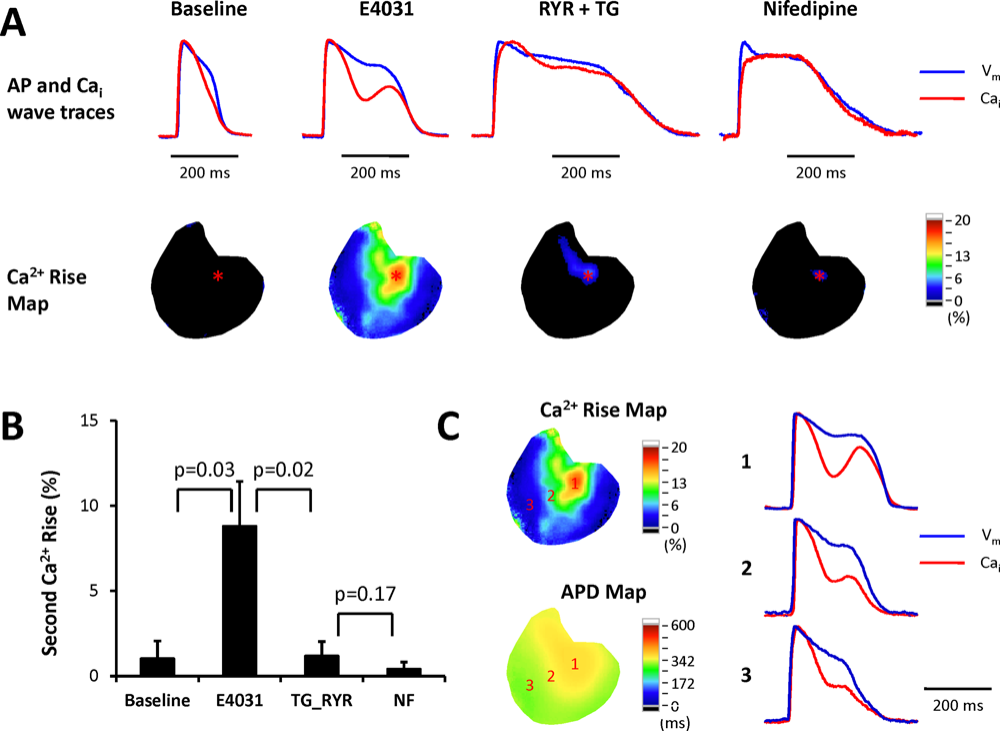
Secondary Ca_i_ rise. **A.** Representative secondary Ca_i_ rise traces and secondary Ca_i_ rise maps. Upper subpanels show the simultaneous recording of V_m_ and Ca_i_ traces. Lower subpanels are the secondary Ca_i_ rise maps. Ryanodine plus thapsigargin suppressed secondary Ca_i_ rise. **B.** Mean secondary Ca_i_ rise. **C.** Simultaneous action potential and Ca_i_ wave traces at different sites. The amplitude of secondary Ca_i_ rise was positively correlated with APD.

Previously we reported that apamin induced secondary Ca_i_ rise and EADs in failing hearts [5]. Optical mapping recording showed that EADs were initiated at the area with secondary Ca_i_ rise. Therefore, we analyzed the amplitude of secondary Ca_i_ rise and the relationship between secondary Ca_i_ rise and TdP inducibility. Fig 3A shows representative V_m_-Ca_i_ traces and secondary Ca_i_ rise maps. Significant secondary Ca_i_ rise developed at the basal area of the left ventricle in the presence of E4031. The amplitude of secondary Ca_i_ rise was positively correlated with the APD with E4031. Fig 3B shows the average secondary Ca_i_ rise at baseline, in the presence of E4031, further administration of ryanodine plus thapsigargin, and then nifedipine. At baseline, there was only minimal secondary Ca_i_ rise (1.0 ± 1.0%), which was significantly enhanced by E4031 (8.8 ± 2.6%, p = 0.03). Ryanodine plus thapsigargin prolonged APD but significantly suppressed secondary Ca_i_ rise (1.2 ± 0.9%, p = 0.02). Nifedipine partially restored the extremely prolonged APD and further suppressed secondary Ca_i_ rise (0.4 ± 0.4%). As shown in Fig 3C, the highest secondary Ca_i_ rise site presented the longest APD (point 1), and vice versa, the amplitude of secondary Ca_i_ rise was less obvious at the shorter APD area (point 3). Fig 1D shows the relationship between TdP inducibility and the amplitude of secondary Ca_i_ rise in each heart. The filled bars indicate hearts with inducible TdP and the unfilled bars are hearts without TdP. Note the 3 non-inducible hearts had relatively lower amplitude, although the animal number was too small for statistical testing.

**Fig 4:**
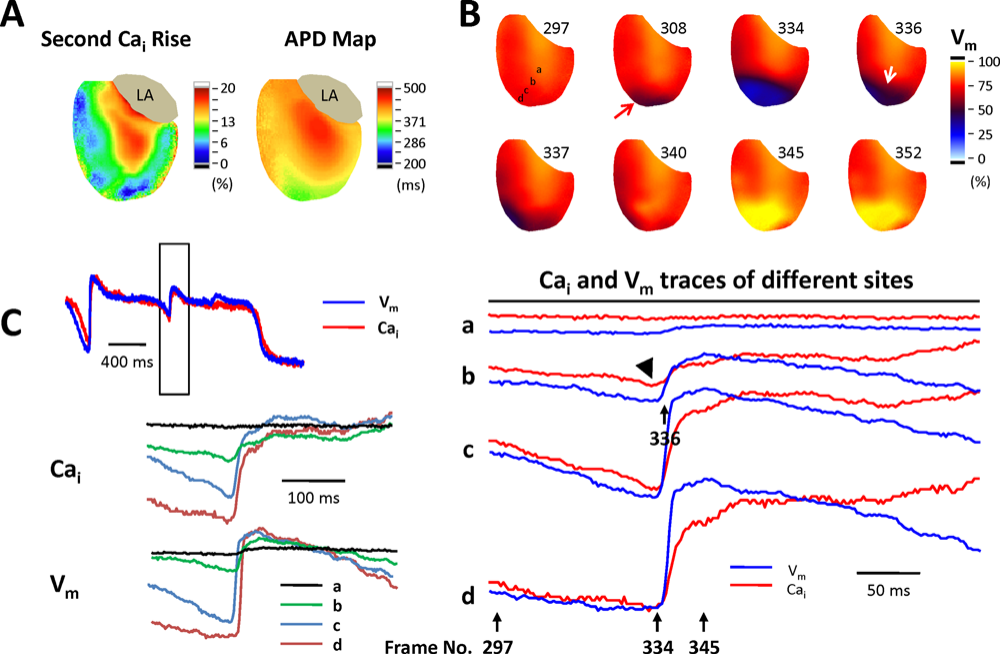
A representative example of the initiation of an early afterdepolarizations (EAD). **A.** Secondary Ca_i_ rise and APD maps show the association of amplitude of secondary Ca_i_ rise and APD. **B.** Serial V_m_ maps show the initiation of the EAD. The numbers indicate the frame numbers. Repolarization developed from the apex (red arrow) toward middle LV (frame 308), and then the EAD initiated from the site b (frame 336, white arrow). **C.** Left subpanels are simultaneous action potential and Ca_i_ traces at different sites (the alphabets indicate the corresponding sites in secondary Ca_i_ rise and APD map of panel B). When merging V_m_ and Ca_i_ traces of the sites, there was Ca_i_ pre-fluorescence at frame 336 (black arrowhead).

Fig 4 shows an example of EAD mapped optically. Panel A shows the secondary Ca_i_ rise and APD maps. The greater-secondary Ca_i_ rise area co-localized with the longer-APD area. Panel B shows serial V_m_ maps of initiation of an EAD (the numbers indicate the frame). Frame 308 shows that repolarization developed from the apex (red arrow) toward the middle LV. Frame 334 shows that the basal area sustained at plateau phase (site a, the red color indicating high V_m_) and the apical area developed repolarization (site c and d, the blue color indicating low V_m_). Frame 336 shows that the earliest activation sites of this EAD beat was at the border of the high secondary Ca_i_ rises region (site b, white arrow), propagating to the apical area (dashed white arrow, frame 337). Panel C shows the corresponding V_m_ and Ca_i_ traces of this particular EAD in panel B. The bottom and right V_m_ and Ca_i_ traces were zoomed-in signals of the black box in the top subpanel, representing the V_m_ and Ca_i_ signals of the corresponding sites on the phase map. Note that the Ca_i_ trace (red lines) shows earlier Ca_i_ pre-fluorescence (black arrow head) at site b, which co-localized with the initiation of the EAD in the V_m_ map (Fig 4C, right subpanels).

### Calcium decay

**Fig 5:**
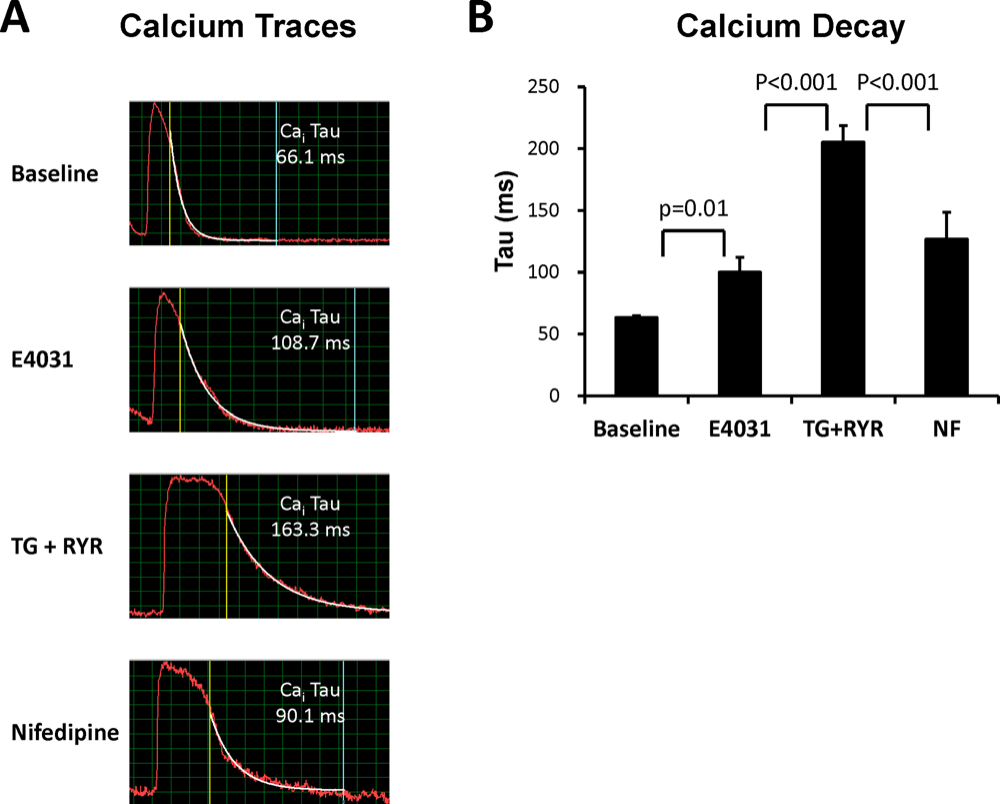
Calcium decay. **A.** A representative example of Ca_i_ (tau value). **B.** Mean Ca_i_ decay.

Whether calcium decay is important in the development of TdP is not clear. Fig 5A shows a representative example of the calcium decay at baseline, in the presence of E4031, further administration of ryanodine plus thapsigargin, and then nifedipine. The trend of calcium decay was similar to the trend of APD_80_. The average calcium decay were significantly prolonged by E4031 (Fig 5B, from 63 ± 1 ms to 100 ± 12 ms, p = 0.01) and then further prolonged after administration of ryanodine plus thapsigargin (205 ± 13 ms, p < 0.001). Nifedipine shortened the calcium decay (126 ± 22 ms, p < 0.001). Similarly, although inhibition of SR Ca^2+^ cycling further delayed the calcium decay, the inducibility of TdP was not further enhanced.

### Arrhythmia Pattern of Torsades de pointes

**Fig 6:**
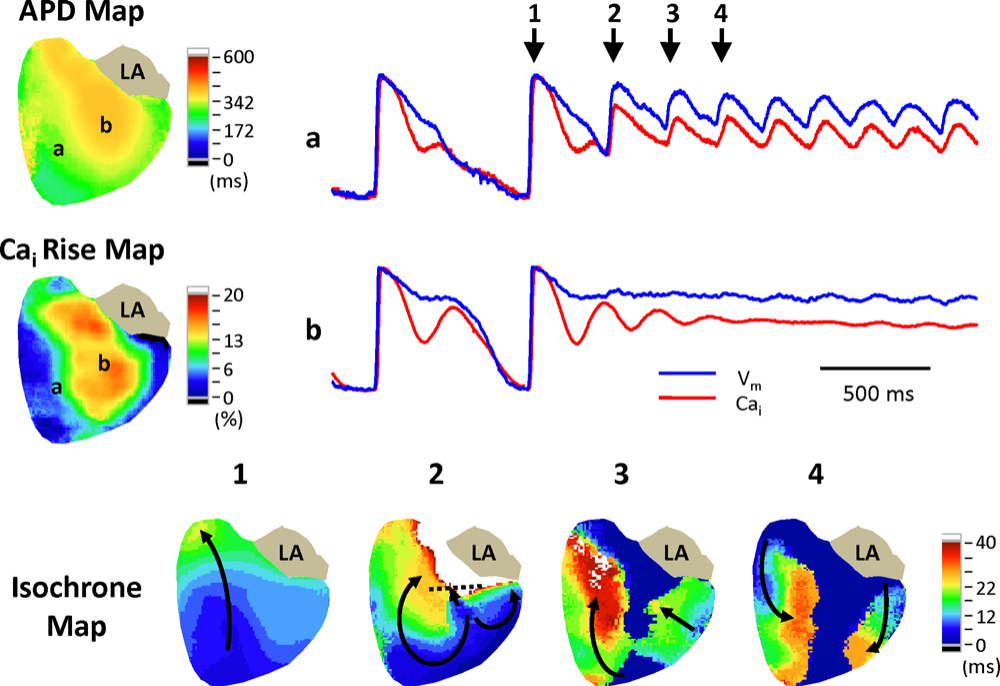
Arrhythmia pattern of TdP. The right top subpanel shows V_m_ and Ca_i_ traces of the corresponding sites in the APD and Ca_i_ rise maps. The isochrone maps show the EAD beat (beat 2) initiated focally from the border of the “hill” with the longest APD and largest Ca_i_ rise shortly after the intrinsic beat (beat 1) with functional conduction block (black dashed line), followed by reentry (beat 2) and beat to beat changes in wave propagation pattern of beats 3 and 4.

In this long-QT syndrome model, the TdP ventricular tachyarrhythmias usually resulted from beat to beat changes in wave propagation patterns initiated by EADs from the border of the largest Ca_i_ rise area. Fig 6 shows an example of an episode of TdP following an intrinsic escape beat (beat 1). The beat 2 was an EAD beat initiated from the border of the largest secondary Ca_i_ rise region. Because of significant spatially heterogeneous prolongation of APD, the short-coupled EAD beat led to unidirectional conduction block and formed reentry. This arrhythmia pattern is consistent with a previous study reported by Asano et al [11].

## Discussion

The major findings of this study include that inhibition of SR Ca^2+^ cycling suppressed the inducibility of TdP. In this model, inhibition of SR Ca^2+^ cycling did not shorten APD. The mechanism could be the suppression of secondary Ca_i_ rise. We also demonstrated that EADs was initiated from border of the high secondary Ca_i_ rises region with earlier Ca_i_ pre-fluorescence. These results indicate that both SR Ca^2+^ cycling and I_Ca,L_ are important in development of secondary Ca_i_ rise and EADs. Therfore, secondary Ca_i_ rise might be a marker of torsadogenesis risk.

### Effects of SR Ca^2+^ cycling inhibitors on APD

The interaction between APD and Ca^2+^ dynamics is complicated. APD and Ca_i_ are bidirectionally coupled in cardiac tissue: a longer APD usually triggers a larger Ca^2+^ release and a larger Ca^2+^ release can either shortens or prolongs APD [12,13]. A larger Ca^2+^ release enhances Na^+^-Ca^2+^ exchange current to prolong APD, but it also potentiates Ca^2+^ induced I_Ca,L_ inactivation and enhances Ca^2+^-sensitive K^+^ and Cl^-^ currents to shorten APD. Ryanodine has been shown to slow down I_Ca,L_ inactivation through blockade of SR Ca^2+^ release [14]. Enhanced RyR phosphorylation is associated the development of EADs in a long QT 2 rabbit model [15]. The reduced Ca^2+^ release leads with slow inactivation of I_Ca,L_ leads to longer APD. Thapsigargin had been also shown to prolong APD through depletion of SR Ca^2+^, and patch clamp recording showed an increased total influx of Ca^2+^ with a longer duration in the presence of thapsigargin [16]. Therefore, the net effects of SR Ca^2+^ cycling blockade include prolongation of APD, slowing down I_Ca,L_ inactivation and SR Ca^2+^ reuptake.

### The importance of SR Ca^2+^ cycling on EADs genesis

The chain of Ca^2+^ cycling includes I_Ca,L_, Na^+^-Ca^2+^ exchange current and SR Ca^2+^ release and reuptake. I_Ca,L_ reactivation plays a central role in the development of EADs and TdP, and inhibition of I_Ca,L_ is one of the preventive and therapeutic approaches to TdP [6,17,18]. Experimental and simulation studies have shown that I_Ca,L_ may reactivate and reversely repolarize under the situation of reduced outward currents and/or increased inward currents [17]. A recent report showed that blockade of Na^+^-Ca^2+^ exchange current suppresses genesis of EADs without interfering the reactivation of I_Ca,L_ in a H_2_O_2_ oxidative cell model [18]. It suggests that Na^+^-Ca^2+^ exchange current may also play a role in EAD genesis. In this study, our data showed that SR Ca^2+^ cycling inhibition suppressed EAD genesis even in the situation of longer APD. A possible mechanism is that the inhibition of SR Ca^2+^ cycling leads to slowing of Ca^2+^-dependent I_Ca,L_ inactivation, which reduces the possibility of available L-type Ca^2+^ channels for reactivation and the degree of secondary rise of Ca_i_ to attenuate EAD genesis. This study also confirms previous findings that any interference among the Ca^2+^ cycling affects the genesis of TdP [19]. On the basis of this study and previous reports, we postulate that the interaction of L-type calcium channel, SR Ca^2+^ release-reuptake and Na^+^-Ca^2+^ exchanger is required to generate EADs.

### Mechanisms of secondary Ca_i_ rise

The formation of secondary Ca_i_ rise is complicated. Priori et al. first proposed that abnormal Ca^2+^ cycling can be the mechanism of EADs [20]. Piacentino et al. observed Ca^2+^ influx during late portions of action potential in failing human cardiomyocytes using a voltage-clamp model [21]. Zeng and Rudy demonstrated that recovery and reactivation of I_Ca,L_ is the mechanism of EADs [4]. It seems that I_Ca,L_ plays the most important role in genesis of EADs. However, whether or not I_Ca,L_ is the only factor in the EAD genesis was not very clear. Qu et al. investigated the mechanisms of EADs and ultra-long APD using a Luo and Rudy simulation model [22]. In the model, the formation of EADs and ultra-long APD is associated with alteration of window I_Ca,L_, speed of I_K_ activation, slope of the steady-state inactivation curve of I_Ca,L_ and pedestal I_Ca,L_ [22]. SR Ca^2+^ release or reuptake/extrusion can affect window I_Ca,L_, I_K_ activation, inactivation of I_Ca,L_ through Ca^2+^-dependent I_Ca,L_ inactivation, modulating V_m_ by Na^+^-Ca^2+^ exchange current, regulating I_Ks_ and Ca^2+^ activated potassium and chloride currents. The mechanisms of secondary Ca_i_ rise involve the interaction of I_Ca,L_, SR Ca^2+^ cycling and Na^+^-Ca^2+^ exchange current: SR Ca^2+^ release causes inactivates I_Ca,L_; then either sustained high V_m_ or spontaneous Ca^2+^ release-induced depolarization through Na^+^-Ca^2+^ exchange current reactivates I_Ca,L_ [19]. SR Ca^2+^ cycling blockade reduces Ca^2+^ release, subsequently slows I_Ca,L_ inactivation to reduce secondary Ca_i_ rise.

### Arrhythmia pattern of TdP

Most EADs initiated from the border zone rather than the region with largest amplitude of Ca_i_ rise in this study and the previous studies [5]. The mechanism of this phenomenon is not clear, and the reason could be the long refractory period of the myocardial tissue with the longest APD. Amplitude of Ca_i_ rise is correlated with the APD, and the excessive prolongation of APD prevents immediate re-initiation of an action potential. The nearby myocardium with relatively shorter APD is available to initiate an EAD. Prolonged APD also leads to unidirectional block during the episodes of TdP. Therefore, the pattern of arrhythmias was focal trigger of EADs and reentry due to heterogeneously prolonged APD in this model.

### Clinical implications

Prolongation of QT interval leading to TdP is one of the major adverse effects of medications. It has been reported that the risk of drug-induced QT prolongation and TdP is associated with I_Kr_ blockade activity at the therapeutic level [23]. The concern leads to significant amount of drugs being withdrawn from the market or even never entering the market [24]. Some medications prolong QT interval but carry relatively low risk of TdP, such as amiodarone. Amiodarone has been reported to affect L-type Ca^2+^ channel and SR Ca^2+^ cycling [25,26]. It is possible that the effects of amiodarone on Ca^2+^ homeostasis lead to anti-arrhythmic property with relatively less torsadogenic effects. The mechanisms can be explained partially by the effects of SR Ca^2+^ blockade on suppressing inducibility of TdP. SR Ca^2+^ homeostasis can also be one of the targets to manage TdP clinically.

### Limitations

There are still some limitations in this study. We mapped only the epicardium of hearts and were not able to recognize some arrhythmias when EADs initiated from mid-myocardium, sub-endocardium or outside of mapping field. Purkinje-ventricular escape rhythm after AV node ablation depended on the level of ablation, and the escape rate was different among the hearts. Because of cardiac memory, the rate of escape rhythm might affect APD and the inducibility of TdP.

## Conclusion

In this AVB and long QT rabbit model, inhibition of SR Ca^2+^ cycling reduces the inducibility of TdP. The mechanism might be suppression of secondary Ca_i_ rise, although inhibition of SR Ca^2+^ cycling does not shorten APD. Nifedipine further inhibits the inducibility of TdP. These results indicate that both SR Ca^2+^ cycling and I_Ca,L_ are important in EAD genesis.

## References

[1] Hoffmann P, Warner B. Are hERG channel inhibition and QT interval prolongation all there is in drug-induced torsadogenesis? A review of emerging trends. J Pharmacol Toxicol Methods. 2006;53: 87–105. 16289936 10.1016/j.vascn.2005.07.003

[2] Roden DM. Drug-induced prolongation of the QT interval. N Engl J Med. 2004;350: 1013–1022. 14999113 10.1056/NEJMra032426

[3] Moss AJ. Long QT Syndrome. JAMA. 2003;289: 2041–2044. 12709446 10.1001/jama.289.16.2041

[4] Zeng J, Rudy Y. Early afterdepolarizations in cardiac myocytes: mechanism and rate dependence. Biophys J. 1995;68: 949–964. 7538806 10.1016/S0006-3495(95)80271-7 PMC1281819

[5] Chang PC, Hsieh YC, Hsueh CH, Weiss JN, Lin SF, Chen PS. Apamin induces early afterdepolarizations and torsades de pointes ventricular arrhythmia from failing rabbit ventricles exhibiting secondary rises in intracellular calcium. Heart Rhythm. 2013;10: 1516–1524. 10.1016/j.hrthm.2013.07.003 23835258 PMC3832504

[6] Milberg P, Fink M, Pott C, Frommeyer G, Biertz J, Osada N, et al Blockade of I(Ca) suppresses early afterdepolarizations and reduces transmural dispersion of repolarization in a whole heart model of chronic heart failure. Br J Pharmacol. 2012;166: 557–568. 10.1111/j.1476-5381.2011.01721.x 22013922 PMC3417488

[7] Maruyama M, Lin SF, Xie Y, Chua SK, Joung B, Han S, et al Genesis of phase 3 early afterdepolarizations and triggered activity in acquired long-QT syndrome. Circ Arrhythm Electrophysiol. 2011;4: 103–111. 10.1161/CIRCEP.110.959064 21078812 PMC3045276

[8] Choi BR, Burton F, Salama G. Cytosolic Ca2+ triggers early afterdepolarizations and Torsade de Pointes in rabbit hearts with type 2 long QT syndrome. J Physiol. 2002;543: 615–631. 12205194 10.1113/jphysiol.2002.024570 PMC2290501

[9] Chang PC, Wo HT, Lee HL, Wen MS, Chou CC. Paradoxical effects of KB-R7943 on arrhythmogenicity in a chronic myocardial infarction rabbit model. J Cardiol. 2014. 10.1016/j.jjcc.2014.08.002 25241015

[10] Chou CC, Chang PC, Wen MS, Lee HL, Chu Y, Baba A, et al Effects of SEA0400 on arrhythmogenicity in a Langendorff-perfused 1-month myocardial infarction rabbit model. Pacing Clin Electrophysiol. 2013;36: 596–606. 10.1111/pace.12091 23380010

[11] Asano Y, Davidenko JM, Baxter WT, Gray RA, Jalife J. Optical mapping of drug-induced polymorphic arrhythmias and torsade de pointes in the isolated rabbit heart. J Am Coll Cardiol. 1997;29: 831–842. 9091531 10.1016/s0735-1097(96)00588-8

[12] Weiss JN, Karma A, Shiferaw Y, Chen PS, Garfinkel A, Qu Z. From pulsus to pulseless: the saga of cardiac alternans. Circ Res. 2006;98: 1244–1253. 16728670 10.1161/01.RES.0000224540.97431.f0

[13] Goldhaber JI, Xie LH, Duong T, Motter C, Khuu K, Weiss JN. Action potential duration restitution and alternans in rabbit ventricular myocytes: the key role of intracellular calcium cycling. Circ Res. 2005;96: 459–466. 15662034 10.1161/01.RES.0000156891.66893.83

[14] Lacampagne A, Caputo C, Argibay J. Effect of ryanodine on cardiac calcium current and calcium channel gating current. Biophys J. 1996;70: 370–375. 8770213 10.1016/S0006-3495(96)79580-2 PMC1224935

[15] Terentyev D, Rees CM, Li W, Cooper LL, Jindal HK, Peng X, et al Hyperphosphorylation of RyRs Underlies Triggered Activity in Transgenic Rabbit Model of LQT2 Syndrome. Circ Res. 2014;115: 919–928. 10.1161/CIRCRESAHA.115.305146 25249569 PMC4406222

[16] Takamatsu H, Nagao T, Ichijo H, Adachi-Akahane S. L-type Ca2+ channels serve as a sensor of the SR Ca2+ for tuning the efficacy of Ca2+-induced Ca2+ release in rat ventricular myocytes. J Physiol. 2003;552: 415–424. 14561825 10.1113/jphysiol.2003.050823 PMC2343391

[17] Qu Z, Xie LH, Olcese R, Karagueuzian HS, Chen PS, Garfinkel A, et al Early afterdepolarizations in cardiac myocytes: beyond reduced repolarization reserve. Cardiovasc Res. 2013;99: 6–15. 10.1093/cvr/cvt104 23619423 PMC3687754

[18] Zhao Z, Wen H, Fefelova N, Allen C, Baba A, Matsuda T, et al Revisiting the ionic mechanisms of early afterdepolarizations in cardiomyocytes: predominant by Ca waves or Ca currents? Am J Physiol Heart Circ Physiol. 2012;302: H1636–1644. 10.1152/ajpheart.00742.2011 22307670 PMC3330805

[19] Driessen HE, Bourgonje VJ, van Veen TA, Vos MA. New antiarrhythmic targets to control intracellular calcium handling. Neth Heart J. 2014;22: 198–213. 10.1007/s12471-014-0549-5 24733689 PMC4016334

[20] Priori SG, Corr PB. Mechanisms underlying early and delayed afterdepolarizations induced by catecholamines. Am J Physiol. 1990;258: H1796–1805. 2163219 10.1152/ajpheart.1990.258.6.H1796

[21] Piacentino V 3rd, Weber CR, Chen X, Weisser-Thomas J, Margulies KB, Bers DM, et al Cellular basis of abnormal calcium transients of failing human ventricular myocytes. Circ Res. 2003;92: 651–658. 12600875 10.1161/01.RES.0000062469.83985.9B

[22] Qu Z, Chung D. Mechanisms and determinants of ultralong action potential duration and slow rate-dependence in cardiac myocytes. PLoS One. 2012;7: e43587 10.1371/journal.pone.0043587 22952713 PMC3428352

[23] Redfern WS, Carlsson L, Davis AS, Lynch WG, MacKenzie I, Palethorpe S, et al Relationships between preclinical cardiac electrophysiology, clinical QT interval prolongation and torsade de pointes for a broad range of drugs: evidence for a provisional safety margin in drug development. Cardiovasc Res. 2003;58: 32–45. 12667944 10.1016/s0008-6363(02)00846-5

[24] Pollard CE, Abi Gerges N, Bridgland-Taylor MH, Easter A, Hammond TG, Valentin JP. An introduction to QT interval prolongation and non-clinical approaches to assessing and reducing risk. Br J Pharmacol. 2010;159: 12–21. 10.1111/j.1476-5381.2009.00207.x 20141516 PMC2823347

[25] Afanas'ev SA, Lukavskaya IA, Kandinskii ML, Medvedev MA. Effect of amiodarone on functional state of sarcoplasmic reticulum in rat myocardium. Bull Exp Biol Med. 2002;133: 205–207. 12360330 10.1023/a:1015832710818

[26] Hancox JC. Amiodarone blocks L-type calcium current in single myocytes isolated from the rabbit atrioventricular node. Gen Pharmacol. 1997;29: 429–435. 9378251 10.1016/s0306-3623(96)00465-x

